# Supplementary figures and images for: Feasibility of non-contact cardiorespiratory monitoring using impulse-radio ultra-wideband radar in the neonatal intensive care unit
Source: PLoS One. 2020 Dec 28;15(12):e0243939. doi: 10.1371/journal.pone.0243939 (PMC7769476; doi:10.1371/journal.pone.0243939)

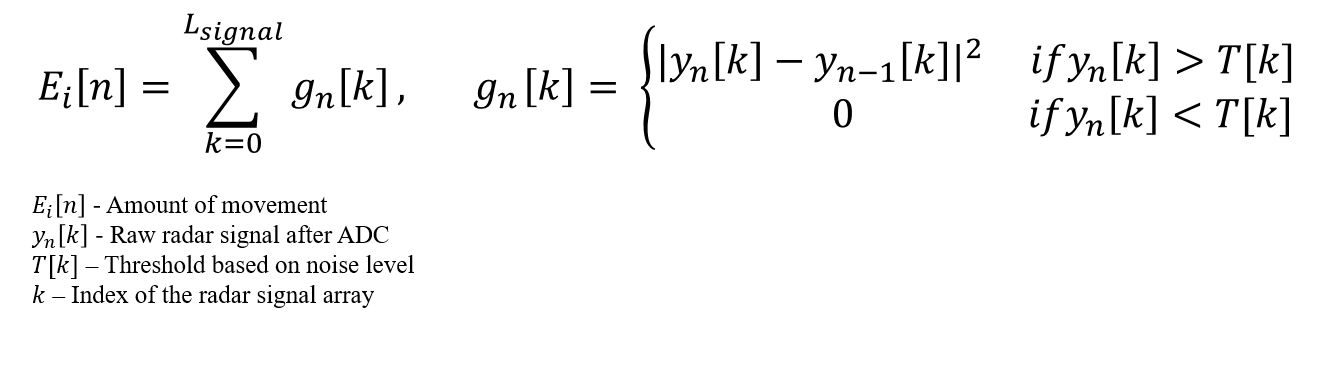


**S1 Data**

Supplement: S1 Data — This approach is suitable for quantifying and assessing an infant's movements because the more the infant moves, the greater the continuous change in signals received from the radar. This method is also used to measure sedentary movement without any change in position [25]. (DOCX) [file pone.0243939.s002.docx]

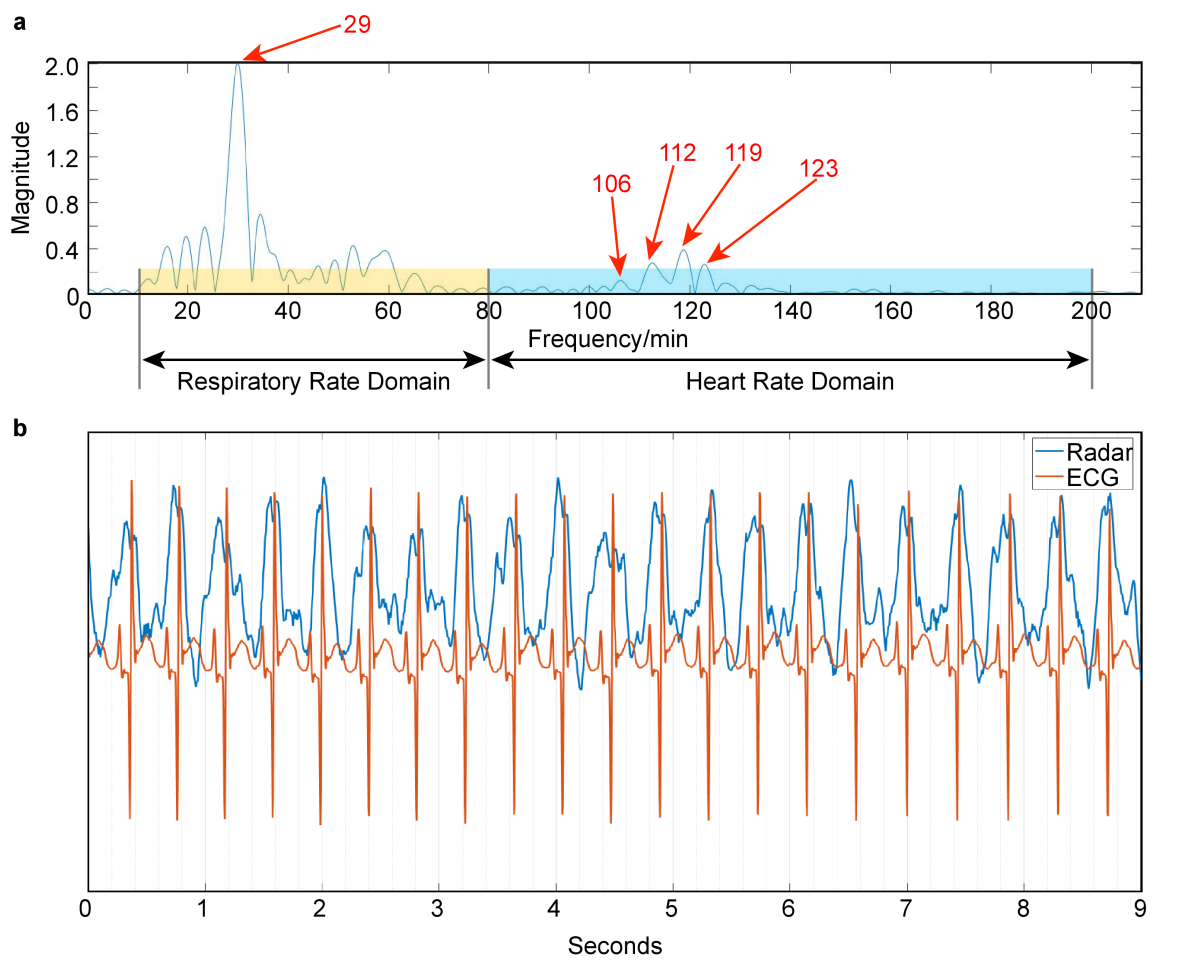


**S1 Fig**

Supplement: S1 Fig — (A) The waveform with the highest magnitude represents the RR frequency location in the spectrum. To extract the HR components, RR harmonic components were removed using the notch filter. Because the magnitudes of RR harmonic components decrease exponentially, the magnitude of the 3rd harmonic component was negligible compared to that of the HR frequency component. (B) Heartbeat waveforms from IR-UWB radar and ECG. The signal waveforms from heartbeats correspond well with the R wave of ECG waveforms. (DOCX) [file pone.0243939.s003.docx]

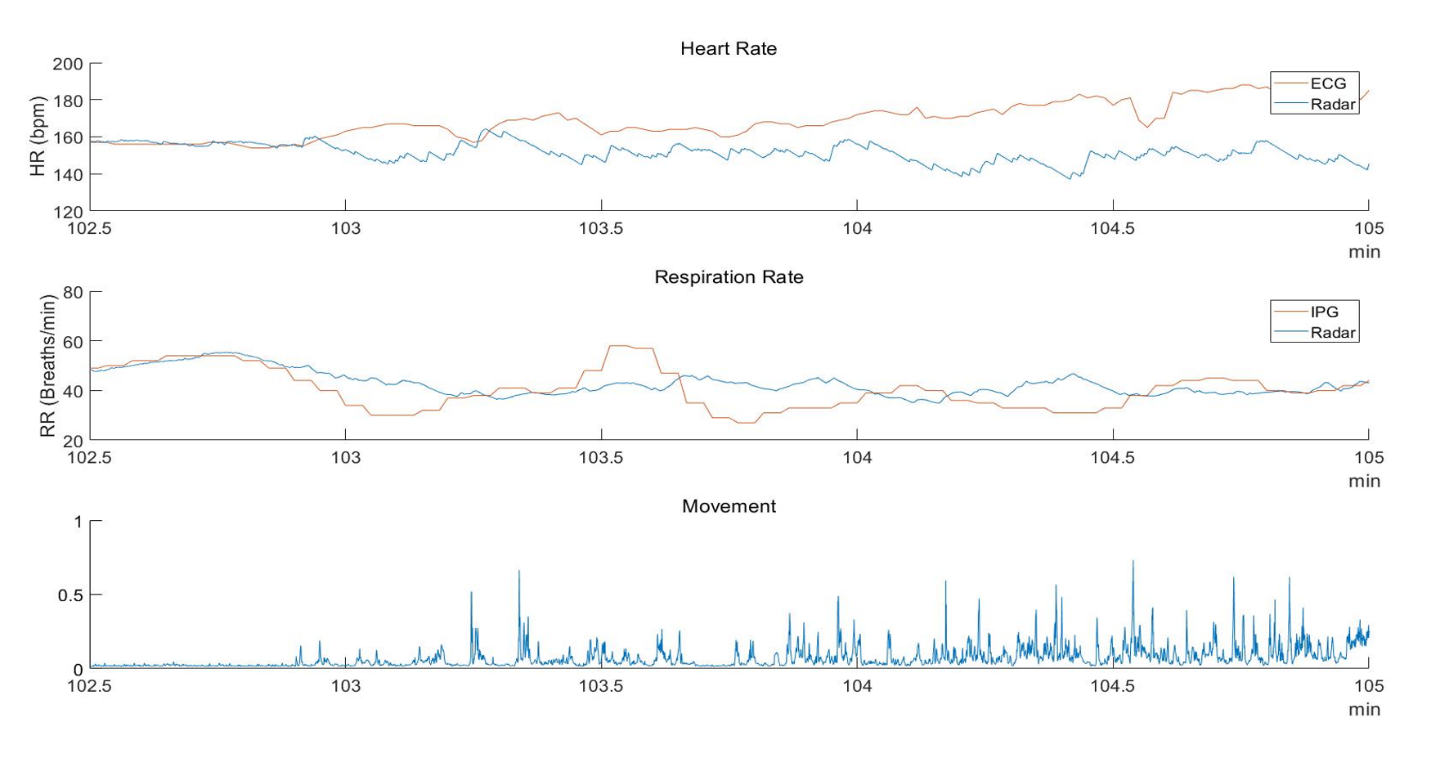


**S2 Fig**

Supplement: S2 Fig — The blue and red lines represent measurements from the radar (RRRd, HRRd) and ECG/IPG (RRIPG, HRECG) over 2.5 minutes, respectively. The degree of movement is presented with arbitrary units based on the distance from the IR-UWB radar (the lowest panel). The HR and RR values of the two sensors differ significantly when notable movement occurs. (DOCX) [file pone.0243939.s004.docx]

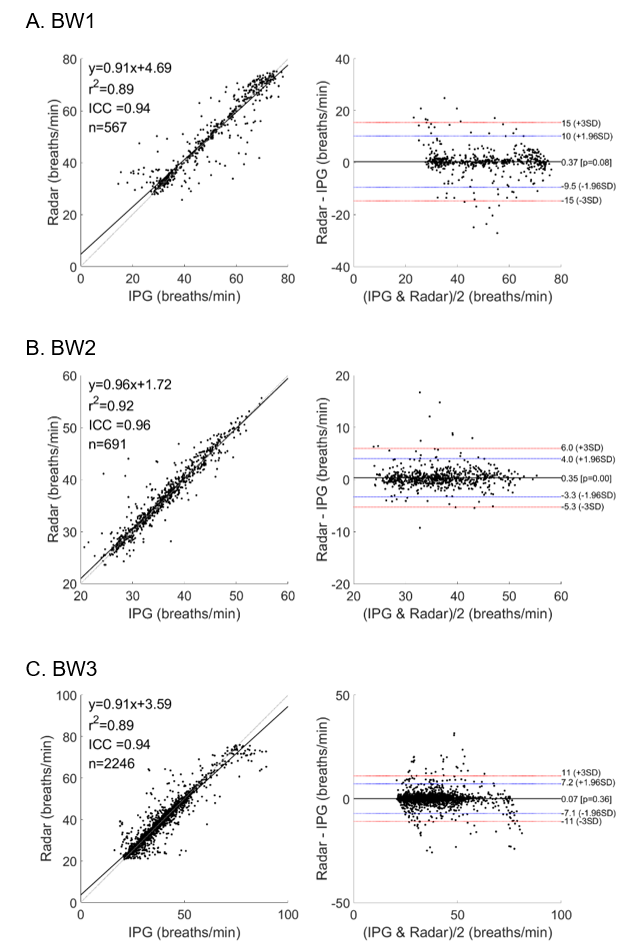


**S3 Fig**

Supplement: S3 Fig — The RRRd shows strong agreement with the RRIPG regardless of body weight. Pearson’s correlation coefficient is shown in the left panel of each graph, and the mean difference and the lower and upper limits of agreement by Bland-Altman plots are indicated by the two blue (±1.96 SD) or red (±3 SD) dotted lines in the right panel of each graph. (DOCX) [file pone.0243939.s005.docx]

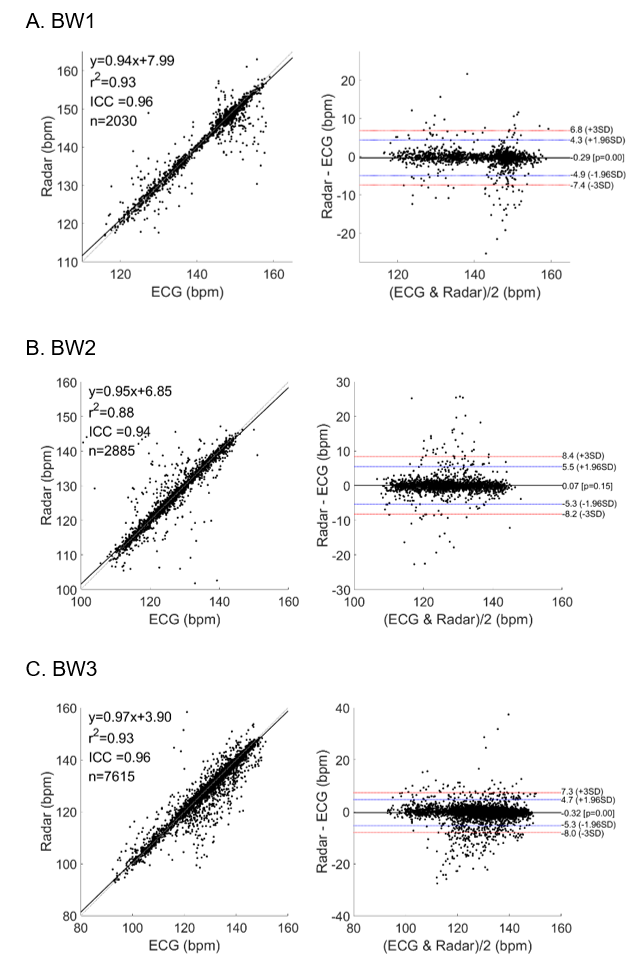


**S4 Fig**

Supplement: S4 Fig — The HRRd also agreed well with the HRECG regardless of body weight. Pearson’s correlation coefficient is shown in the left panel of each graph, and the mean difference and the lower and upper limits of agreement by Bland-Altman plota are indicated by the two blue (±1.96 SD) or red (±3 SD) dotted lines in the right panel of each graph. (DOCX) [file pone.0243939.s006.docx]
