# Supplementary material for: Feasibility of non-contact cardiorespiratory monitoring using impulse-radio ultra-wideband radar in the neonatal intensive care unit
Source: PLoS One. 2020 Dec 28;15(12):e0243939. doi: 10.1371/journal.pone.0243939 (PMC7769476; doi:10.1371/journal.pone.0243939)
Supplement: S1 Table — (DOCX) [file pone.0243939.s007.docx]

**S1 Table. Agreement between IR-UWB Radar and ECG/IPG for HR and RR Measurements When the Neonates were Stable: Signal Matching, Correlations, and Differences According to the BW Groups.**

| Patient group | No. of cases/ recordings | Median weight, age at recording (g, d [IQR]) | Recording time (valid/total) (min) | RR detection (RR_Rd_ vs. RR_IPG_) | | | | | HR detection (HR_Rd_ vs. HR_ECG_) | | | | | |
| --- | --- | --- | --- | --- | --- | --- | --- | --- | --- | --- | --- | --- | --- | --- |
|  |  |  |  | No. of samples | CCC [95% CI] | LOA [±3 SD] | Mean bias [95% CI] | p value* | No. of samples | Signal matching (%) | CCC [95% CI] | LOA [±3 SD] | Mean bias [95% CI] | p value* |
| Total | 34/51 | 3,020, 14.5 [7–28] | 22/44 | 3,504 | 0.950 [0.947–0.954] | 7.3, -7.0 [11, -11] | 0.17 [0.05–0.29] | 0 | 12,530 | 95.40 | 0.967 [0.966–0.968] | 4.8, -5.3 [7.5, -8.0] | -0.23 [-0.18– -0.27] | 0 |
| BW1 | 7/7 | 1,880, 21 [15–28] | 14/34 | 567 | 0.944 [0.934–0.952] | 10, -9.5 [15, -15] | 0.37 [-0.04–0.78] | 0.08 | 2,030 | 95.10 | 0.964 [0.961–0.967] | 4.3, -4.9 [6.8, -7.4] | -0.29 [-0.18– -0.39] | 0 |
| BW2 | 9/11 | 2,430, 9 [6–38] | 19/35 | 691 | 0.958 [0.952–0.964] | 4.0, -5.3 [6.0, -5.3] | 0.35 [0.21–0.49] | 0 | 2,885 | 96.30 | 0.940 [0.936–0.944] | 5.5, -5.3 [8.4, -8.2] | 0.07 [-0.02– 0.17] | 0.15 |
| BW3 | 18/33 | 3,525, 17 [7–27] | 26/52 | 2,246 | 0.941 [0.936–0.946] | 7.2, -7.1  [11, -11] | 0.07 [-0.08–0.22] | 0.36 | 7,615 | 95.10 | 0.962 [0.960–0.963] | 4.7, -5.3 [7.3, -8.0] | -0.32 [-0.26– -0.37] | 0 |
