# Supplementary material for: Feasibility of non-contact cardiorespiratory monitoring using impulse-radio ultra-wideband radar in the neonatal intensive care unit
Source: PLoS One. 2020 Dec 28;15(12):e0243939. doi: 10.1371/journal.pone.0243939 (PMC7769476; doi:10.1371/journal.pone.0243939)
Supplement: S1 File — (DOCX) [file pone.0243939.s008.docx]

**
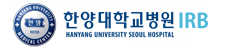
연 구 계 획 서**

| **연구제목** | | | | | | | |
| --- | --- | --- | --- | --- | --- | --- | --- |
| **국문** | 표준방식과 IR-UWB 레이더 기술을 이용한 비침습적 및 비접촉성 신생아 생명징후 모니터링의 정확성 비교 분석 | | | | | | |
| **영문** | **A validation of a non-invasive and non-contact vital sign monitoring method in neonate using IR-UWB radar technique against standard vital sign monitoring methods** | | | | | | |
| 1. **연구자** | | | | | | | |
| **연구책임자** | | **성명** | **박현경** | **소속** | **한양의대** | **직위** | **부교수** |
| **공동연구자** | | **성명** | **이현주** | **소속** | **한양의대** | **직위** | **부교수** |
| **연구담당자** | | **성명** | **김종덕** | **소속** | **한양의대** | **직위** | **임상강사** |
| **연구참여자의 교육 실적** | | | | | | | |
| ◆ 최근 2년 내에 임상연구, 연구윤리 및 GCP에 대한 교육을 이수한 적이 있습니까?  ■ 예 □ 아니오 | | | | | | | |
| **공동연구자/ 연구담당자** | | | | | | | |
| ◆ e-irb 신청서 상에 연구에 참여 예정인 공동연구자 및 연구담당자를 등록해 주시기 바랍니다. 모든 연구자가 등록되었습니까?  ■ 예 □ 아니오  **☞ “아니오”라고 체크한 경우, e-irb신청서의 연구관련자 정보에 모든 공동연구자/연구담당자를 등록하시기 바랍니다.** | | | | | | | |
| 1. **연구계획** | | | | | | | |
| **연구목적** | | | | | | | |
| 레이더를 이용한 비침습적 및 비 접촉성 생명징후 모니터링 방법의 개발 및 그 정확성을 기존 생명징후 모니터링의 표준 검사법이라고 할 수 있는 침습적 또는 접촉성 생명징후 모니터링법과 비교하여 분석하고자 한다. | | | | | | | |
| **배경 및 이론적 근거** | | | | | | | |
| 1. 연구의 필요성   호흡수와 심박수 등과 같은 생명징후(Vital sign)의 모니터링은 지금까지는 주로 병원환경에서 환자들을 대상으로 의학적인 요구에 그 필요가 있어 다양한 측정방법이 개발되어왔다. 이러한 생명징후 모니터링은 근래에 들어서는 병원 이외의 장소에서도 질병 및 건강관리의 측면에서 그 이용빈도가 늘어나고 있으며, 병원 내에서는 주로 신생아 또는 성인 중환자실과 같은 환경에서 정확성을 기하기 위해 침습적 또는 접촉성 방법의 보다 다양한 방법의 생명징후의 모니터링이 이루어지고 있다. 하지만 현재 병원환경에서 이루어지는 접촉 또는 침습적 생명징후 모니터링 방법은 중환자실 내 면역저하 상태 환자의 기회감염 문제, 미숙아에서 접촉성 단자로 인한 피부 벗겨짐 현상 등 여러 부가적인 문제점이 발생할 위험성을 가지고 있다.  최근 산모의 나이가 고령화 되면서 고위험 임산부가 증가하는 추세를 보이고, 이와 동시에 출산율의 감소에도 불구하고 미숙아의 상대적 출생률은 증가하고 있다. 이런 미숙아뿐만 아니라 출생 28일 이내의 신생아들 역시 모세기관지염, 장염, 패혈증 등 다양한 원인으로 신생아중환자실에 입원하게 되며, 이러한 환아들은 무호흡, 서맥, 저혈압 등 불안정한 생명징후에의 노출 위험성이 높아 이를 위해 24시간 생명징후 모니터링의 필요성이 높다. 현재까지 이러한 생명징후 모니터링을 위해 접촉식, 침습식 생명징후 센서를 이용하고 있으나, 이러한 방법은 환아의 움직임으로 인한 거짓 경보가 자주 나타나며 또한 피부 미숙으로 인한 부착 부위 피부 벗겨짐과 괴사, 혈관 순환 장애, 기회감염 등 환아들이 위험에 노출될 가능성이 상시 존재한다.  이러한 이유들로 병원 내원 환자, 특히 본 연구와 관련된 신생아들에게서 기존의 표준방식 모니터링은 침습적, 접촉식 모니터링의 방법으로 인해 그 한계점을 보이고 있으며, 이와 같은 한계점 극복을 위한 새로운 생명징후 모니터링의 방법을 찾아야 할 필요성이 높아지고 있다.   1. IR-UWB 레이더 기술의 배경  - IR-UWB 레이더 기술을 이용한 새로운 생체신호 및 움직임 모니터링 기술   IR-UWB 레이더 기술은 광대역 주파수를 점유하며 시간 축에서 매우 짧은 지속시간을 갖는 임펄스성 신호를 이용하는 기술로 시간 축 분해능이 매우 뛰어나 수면 중 보이는 호흡 및 심박 등으로 인한 미세 움직임 탐지에 매우 유용함.  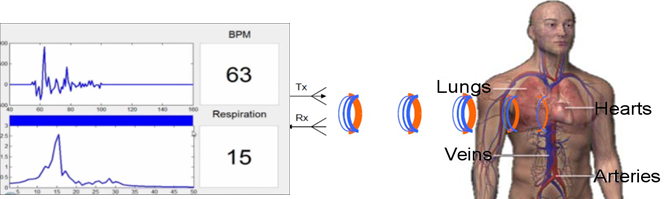   - 실제 IR-UWB 레이더 기술을 이용한 호흡수 및 심박수 측정과 관련된 연구가 세계 각지에서 활발히 진행되고 있으며 이와 관련된 제품 연구 또한 활발히 진행되고 있음. - IR-UWB 레이더 기반의 수면 모니터링 기술은 단일 개의 센서로 호흡, 심박 및 움직임 정도, 무호흡 증세를 탐지 할 수 있으며 혈압까지 측정 할 수 있는 기술적 가능성을 지님. - IR-UWB 레이더 기반의 수면 모니터링 기술은 비접촉/비침습식 방법으로, 원거리에서 비 접촉식으로 피측정자의 생체 신호 정보를 측정 할 수 있음. - 기존의 부착 및 착용류의 수면 검사 장비들과 달리 비접촉/비침습식 원거리 특성을 갖는 센서의 특징으로 인해 수면 모니터링과 관련한 사용자들의 편의성 증대 및 거부감 해소가 가능함. - IR-UWB 레이더 기반의 수면 모니터링 센서는 저가형 및 소형으로 제작 가능하여 사용자로 하여금 부담 없는 가격에 구매 및 사용 가능.   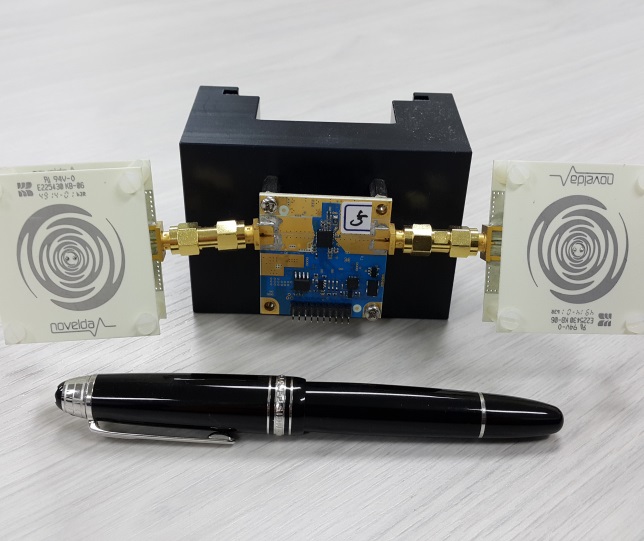   - 단일 개의 센서로 2명 이상의 다수 인원에 대해 동시에 생체 신호 정보를 추출 할 수 있는 기술적 가능성을 지님. - IR-UWB 레이더를 활용한 생체신호 모니터링 센서 활용 시, 병원 내에서 비 접촉식으로 생체 신호 실측 가능 뿐만 아니라 병원이 아닌 가정에서의 활용이 가능하여 신생아, 노약자, 환자 및 거동이 불편한 장애인 등 다양한 조건을 갖는 개인들이 모두 이용 가능. - 다양한 특장점을 갖는 IR-UWB 레이더 기반의 수면 모니터링과 관련하여 한양공대 연구팀은 세계최고 수준의 기술력을 확보하고 있으며, 연구팀의 사전 연구로 인한 연구 성과는 세계적인 영상 공유 사이트인 YouTube에도 게시되어 있으며, 해당 영상은 칩 제조사인 NOVELDA에서 또한 소개하고 있을 정도로 성능을 간접적으로 인정받음.   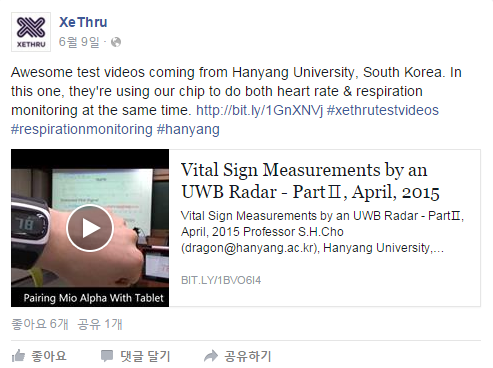  따라서 세계적인 수준의 기술을 보유하고 있는 현 시점에서 실제 병원환경에서 이루어지고 있는 침습적 또는 접촉식 방법을 통한 기존의 표준 방법에 의한 생체 신호 모니터링과 비교하여 IR-UWB 레이더 방법을 통한 생체 신호 모니터링 방법의 정확도를 확인함으로 향후 다양한 환경에서 정확한 생체 신호를 비접촉식으로 얻을 수 있는 기술 및 기기를 개발하는 것은 임상적으로 중요한 의의가 있다고 할 수 있다. | | | | | | | |
| **연구방법** | | | | | | | |
| 1. 연구 범위 및 내용 (방법) 2. 연구개요   연구는 한양대학교 공대 조성호 교수팀과 한양대학교 병원 의료진이 소아청소년과 (신생아중환자실) 에서 기존의 표준 측정장비를 통해 생명징후 (호흡수, 심박수, 혈압)를 모니터링 하는 입원환자들을 대상으로 기존 측정 방법을 통해 측정된 생체 징후와 IR-UWB 레이더를 이용해 측정한 생명징후의 정확성을 비교하는 방법으로 이루어지는 연구이다. 대상군은 IRB를 통과한 시점부터 18개월동안 신생아중환자실에 입원한 재태기간 37주 이상의 만삭아로 선정하며, 이들을 대상으로 생명징후의 지속 및 반복 측정을 통하여 정확도를 보고자 한다.  본 연구의 임상적 1차 지표는 분당 심박수(beats/min) 및 분당 호흡수(respiration rate; cycles/min)의 기존의 심전도를 이용한 심박동 측정방법 및 고식적 호흡수 측정 방법과 본 연구에서 측정된 IR-UWB 레이더를 이용한 측정방법 사이의 interclass correlation 을 제시하고자 하는 것이다. 이를 통해 IR-UWB 레이더를 이용한 새로운 방식의 생명징후 모니터링이 기존의 방식과 비교하여서 정확한 결과값을 보일 수 있을 것임을 기대한다.  측정 지표는 참여자당 신생아중환자실 입실 기간 중 IR-UWB 레이더와 기존 방법(심박수의 경우 ECG, 호흡수의 경우 고식적인 육안 측정 및 pulse oximeter, sensing device) 으로 동시에 측정된 심박수와 호흡수이다.   1. 연구의 구체적 방법  - 임상실험 환경 구축 - 한양대학교 의과대학 서울병원과 협력하여 테스트베드 환경을 구축. - 신생아중환자실 입원 환아의 생명징후 모니터링을 위하여 신생아중환자실 내 신생아용 인큐베이터 및 바구니 상단(약 1.8m)에 상시 IR-UWB 레이더 신호 계측 가능한 환경을 구축함.   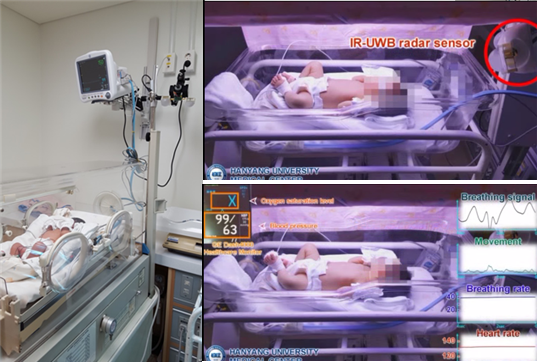  < 한양대학교 신생아중환자실 내 시험 구축한 테스트베드 환경 >   - 호흡 / 심박수 추출 알고리즘 개선   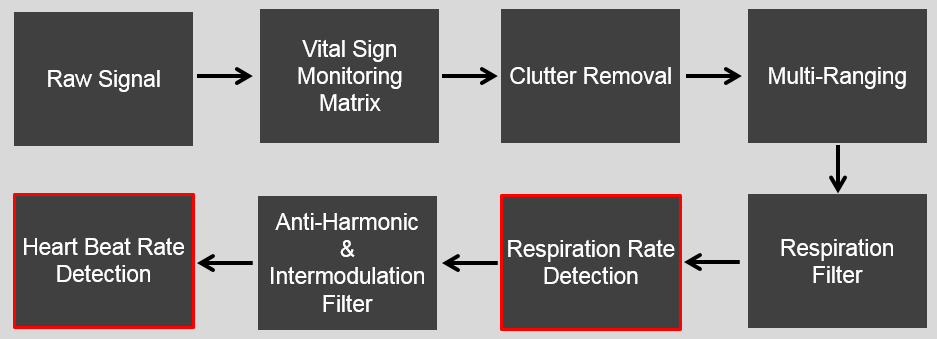  < 호흡수 및 심박수 측정 알고리즘 블록도 >   - 호흡수 측정 알고리즘의 안정성 향상을 위하여, IR-UWB 레이더 신호 내, 다중 포인트를 후보 점으로 하여 신뢰성이 가장 높은 포인트의 호흡수 정보를 추출함.   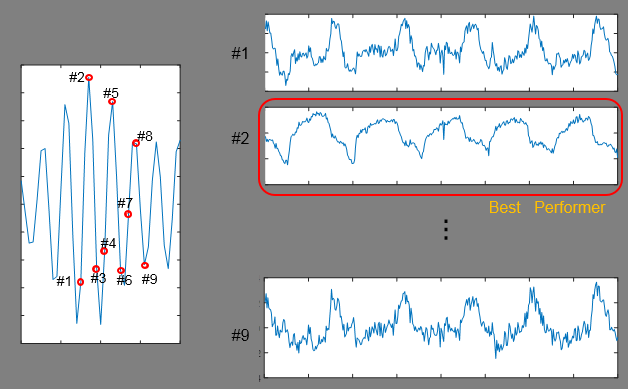  < 호흡수 측정 정확도 향상을 위한 다중 포인트 후처리 알고리즘 >   - IR-UWB 레이더를 통한 심박수 및 호흡수 측정 - 호흡 뿐만 아니라, 수면 중 심정지, 심박 이상 등의 정보 추출을 위하여 심박수 측정 알고리즘 추가하였으며, 과도한 움직임이 없는 경우에 한하여 비교적 정확함.. - IR-UWB 레이더를 통한 심박 신호 검출 가능성 확인을 위하여, 호흡 정지 후 레이더 파형을 조사함(호흡 신호 성분 제거를 위함).   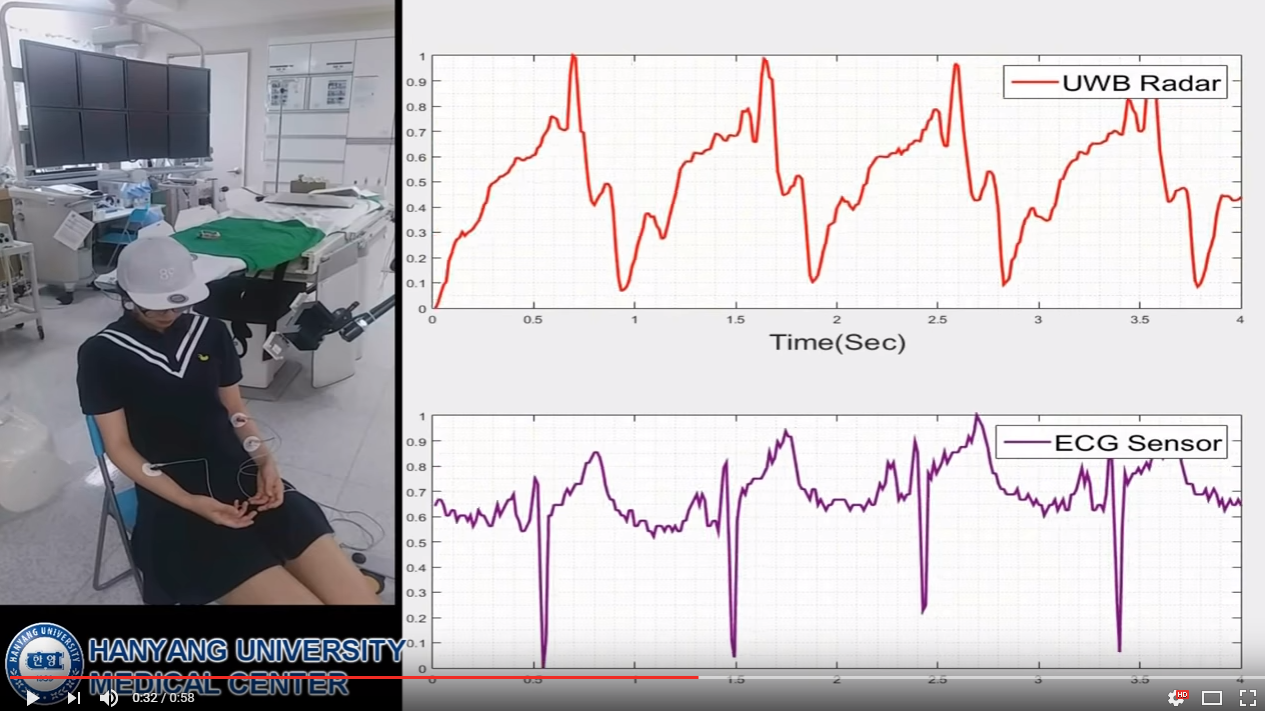  < 26세 여성의 ECG(심전도;아래)와 레이더 신호(위)>   - 임상실험 세부계획 - 연구에 동의 완료 된 대상가능 환아를 신생아중환자실 내 구축된 테스트베드 내 위치 후 기존 방식을 통한 심박수, 호흡수 측정 및 IR-UWB 레이더를 통한 심박수 및 호흡수 측정을 3일동안 지속적으로 측정하여 이에 대한 정보를 데이터베이스화 하여 저장한다. - 이 외, 1일 3회 정해진 시간에 두 방법 모두 모니터에 측정되는 결과를 증례기록지에 기록한다. 이때 환아의 움직임의 유무 및 정도 역시 같이 기록한다. - 본 임상연구의 수행과정에서 환아의 모니터링은 신생아중환자실 내 4면이 밀폐 가능한 격리실 내에서 진행할 계획이며, 이를 통해 신생아 중환자실 내 다른 환아들에게 본 연구 관련 영향이 미치지 않도록 한다.  1. 연구의 기대효과  - 원거리 환자의 상시 모니터링 (비침습적 및 비 접촉식) - 미숙아의 경우 무호흡 증세와 같은 치명적인 상황에서 실시간 알림 기능으로 발빠른 응급 서비스 구현 가능 - 보호자 없이 단독으로 거주하는 노인이나 의료 시설이 갖춰지지 않은 무의촌 환자의 생체 신호를 모니터링 할 수 있어 공무원들의 업무 효율성을 증대시킬 수 있고 고위험군 환자들을 조기에 인지하여 대처할 수 있음  1. 연구추진계획   1) 연구 추진전략 및 방법   - 네트워크 구축을 통한 연구 인프라 형성 - 한양대학교 의료원 내 임상실험 장소와 연구실의 네트워크 구축을 통한 임상 연구의 인프라 형성 및 한양공대 레이더 연구팀과의 연구 인프라 구축 - 전문 의료진과의 협력 - 한양대학교 병원의 신생아 및 소아호흡기 전문의 - 정기 회의를 통한 지속적인 피드백 활용 - 의학적인 관점에서의 데이터 타당성 확보 - 측정 데이터의 의학적인 활용 가능성 확장 - 임상 실험을 통한 검증 - 실제 병원에서 측정된 의학데이터와의 비교  \| 1. 연도별 연구 추진일정 \| \| \| \| \| \| \| \| --- \| --- \| --- \| --- \| --- \| --- \| --- \| \| 연도 \| 연구의 내용 \| 추진일정 \| \| \| \| 비고 \| \| 2017 후반기 \| 2018 전반기 \| 2018 후반기 \| 2019 전반기 \| \| 2017-8년도 \| 생체 신호 모니터링 성능 및 기능 개선 \| ○ \| ○ \| ○ \|  \|  \| \| 2017-8년도 \| 하드웨어 구축 \|  \| ○ \| ○ \|  \|  \| \| 2017-8년도 \| 소프트웨어 구현 \|  \| ○ \| ○ \| ○ \|  \| \| 2017-8년도 \| 임상 실험을 통한 신뢰성 검증 \|  \| ○ \| ○ \| ○ \|  \|   **4. 연구기간**  기관생명윤리위원회 통과 후 2년까지.  **5. 피험자 선정기준, 제외기준, 목표한 피험자수 그 근거**  1) 피험자 선정기준   1. 동의서를 획득한 신생아중환자실 입원 재태주령 37주 이상의 만삭 신생아들 중 생명징후 모니터링이 필요한 환자   2) 피험자 제외기준   1. 연구참여에 동의하지 아니한 환자 2. 연구 시행 후 대상자의 급격한 임상증상의 악화 및 활력징후의 변화로 인해 임상의의 판단으로 연구를 위한 활력징후 측정을 중단하여야 하는 경우   3) 취약한 연구대상자 보호 방법  본 연구의 연구대상자는 만 19세 미만의 미성년자를 포함하고 있어 부모 혹은 기타 법적 보호자의 동의가 필요하다. 따라서 본 연구에서는 취약한 연구대상자의 보호를 위해 만 6세 미만에서는 연구대상자의 법적 보호자에게 서면 동의를 받은 경우에 한해 연구대상에 포함하도록 한다.  4) 목표 피험자수  Statistical analysis software PASS 2008을 이용해 산출하였으며, Primary analysis는 기존의 접촉을 통해 측정한 심박수/호흡수와 IR-UWB 레이더를 이용해 비접촉적 방법으로 측정한 심박수/호흡수 간의 Interclass correlation coefficient R (ICCR) 이다. 활력징후에 관한 검사임으로 정확도가 높은 수준 이여야 함을 고려해서 Power=0.90, alpha=0.01 로 가정하면, 각 지표에 따라 다음과 같다.   1. 심박수 (beats/min): 50 명   본 연구에서 사용하는 통계방법인 Interclass Correlation Coefficient(클래스간 상관관계 계수)법은 일반적으로 상관계수(R)가 0.75이상일시에 Excellent inter-observer variability로 봄에도 불구하고, 본 연구의 주제가 활력징후 인 점을 고려해서 더욱 높은 정확도를 보여야 한다고 판단된다. IR-UWB 레이더를 이용한 방법이 비 접촉적 방법이라는 점에서 획기적이기는 하지만 적어도 최근 활발히 개발되고 있는 Wearable heart rate monitoring device 와 비슷한 수준의 정확도를 보여야 한다. 예로 최근 Gile 등 (*Eur J Appl Physiol (2016) 116:563–571*) 이 보고한 Polar V800 heart rate monitor 의 경우도 ECG와 비고할 때 ICCR 이 0.976에 이르는 매우 높은 수준이었으며, Wallen 등 (*PLoS ONE 2016, 11(5); e0154420*) 의 보고에서도 Apple watch는 0.98, Mio ALPHA는 0.91, Samsung Gear S는 0.80으로 높은 수준이었다. 이에 ICCR을 0.90 ~ 0.98으로 잡았다.  따라서 ICCR 이 <0.90 일때 기존의 측정방법과 비교하여 낮은 정확도를 보일 것으로, ICCR >0.98 일 때 기존의 측정방법보다 우월한 결과를 보일 것으로 가정하여 이를 기준으로 통계적 정확도를 고려하면 최소 21명의 sample size 가 필요할 것으로 판단되며, 결과값 추출의 제한 및 대상자 탈락의 가능성이 높은 소아 대상임을 고려하여 이의 2배 가량인 50명을 목표 피험자수로 설정한다.   1. 호흡수: 50 명   Bergese 등의 *(Anesth Analg 2017;124:1153–1159)* 보고에 따르면 Medtronic 사의 pulse oximetry (NellcorTM OxiMax N-600x Pulse Oximeter) 로 측정한 호흡수는 건강한 사람을 대상으로 시행한 조사에서 0.94 의 ICC를 보이는 것으로 조사되었다. 본 연구에서는 고식적 호흡수 측정 방법을 통해서 측정되는 호흡수 측정방법과 IR-UWB 레이더를 통해서 측정되는 호흡수간의 ICC를 기존 pulse oximeter를 이용해 접촉을 통해 측정하는 방법과 적어도 비슷한 수준으로 정했다. 이에 심박수와 같이 ICCR를 0.90 ~ 0.98로 설정하고 계산하여 50명을 목표 피험자수로 설정한다.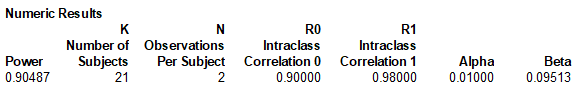  **6. 효과 평가기준, 평가방법 및 해석방법 (통계분석방법)**  평가기준은 IR-UWB레이더로 측정된 심박수/호흡수와 기존 방법 (심박수의 경우는 심전도, 호흡수의 경우는 manual counting 또는 sensing device) 간의 일치도를 보는 것으로 통계 분석 방법은 크게 3가지 방법을 이용하려고 합니다.   1. Simple correlation, linear regression 과 residual plot 2. Interclass correlation coefficient R (ICCR) 3. Lin’s Concordance Correlation Coefficient R (CCC) | | | | | | | |
| **연구예정기간** | | | | | | | |
| **IRB 승인일 이후 ~ 승인 후 2년** | | | | | | | |

- 연구의 윤리적 고려 사항
  - 윤리성 관리기준

본 연구를 수행함에 있어서 연구에 참여하는 모든 참여자들은 헬싱키 선언, 생명윤리 및 안전에 관한 법률 등의 내용을 준수할 것이며, 본원의 표준작업지침서에 정해진 절차를 준수하면서 윤리적이고 과학적으로 시행하겠습니다.

- - 피험자를 위한 설명서 및 피험자 동의서

피험자를 위한 연구 설명서 및 동의서는 별도로 문서화하여 진행되며, 연구 동의 시 연구담당자에 의한 설명과 함께 피험자에게 연구 설명서 및 동의서의 사본이 제공될 것입니다.

- - 비밀보장방법

본 연구에서는 연구 참여자의 신원을 파악할 수 있는 기록은 기밀유지를 원칙으로 하고 있으며, 개인 정보 및 인적사항을 따로 수집하지 않습니다. 따라서 연구진행 과정이나 이후 출판 과정에서 참여자를 식별하는 것이 불가능합니다. 참여자들의 동의서 및 연구 과정에서 수집된 데이터 및 파일들은 Berlin Data Protection Act에 따라서 철저하게 보호될 것이며, 연구목적으로만 사용할 예정이며, 연구를 진행한 한양대학교병원 신생아중환자실 내부의 번호키가 달린 수납장에 보관될 것입니다. 통계 분석, 연구 결과 발표나 출판 시 역시 대상자의 신원을 알 수 있는 정보는 포함되지 않습니다. 이러한 모든 비밀 보장 및 안전관리는 책임 연구자인 소아청소년과 박현경 교수가 담당하여 진행합니다.
