# Supplementary material for: Feasibility of non-contact cardiorespiratory monitoring using impulse-radio ultra-wideband radar in the neonatal intensive care unit
Source: PLoS One. 2020 Dec 28;15(12):e0243939. doi: 10.1371/journal.pone.0243939 (PMC7769476; doi:10.1371/journal.pone.0243939)
Supplement: S2 File — (DOCX) [file pone.0243939.s009.docx]

**
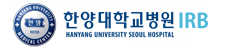
Clinical Trial Protocol**

| **Title** | | | | | | | |
| --- | --- | --- | --- | --- | --- | --- | --- |
| **Korean** | 표준방식과 IR-UWB 레이더 기술을 이용한 비침습적 및 비접촉성 신생아 생명징후 모니터링의 정확성 비교 분석 | | | | | | |
| **English** | **A validation of a non-invasive and non-contact vital sign monitoring method in neonate using IR-UWB radar technique against standard vital sign monitoring methods** | | | | | | |
| 1. **Researchers** | | | | | | | |
| **Research director** | | **Name** | **Hyun-Kyung Park** | **Affiliation** | **Hanyang University College of Medicine** | **Position** | **Associate Professor** |
| **Co-resarcher** | | **Name** | **Hyun Ju Lee** | **Affiliation** | **Hanyang University College of Medicine** | **Position** | **Assistant Professor** |
| **Researcher** | | **Name** | **Jong Deok Kim** | **Affiliation** | **Hanyang University College of Medicine** | **Position** | **Clinical Instructor** |
| **Educational status of research participants** | | | | | | | |
| ◆ Have you completed training in clinical research, research ethics and GCP in the last two years?■ Yes □ No | | | | | | | |
| **Co-researcher/ Researcher** | | | | | | | |
| ◆ On the e-irb application form, please register the co-researchers and research personnel who will be participating in the research. Are all researchers registered?  ■ Yes □ No  **☞ If “No” is checked, please register all co-researchers/researchers in the researcher information of the e-irb application.** | | | | | | | |
| 1. **Research Plans** | | | | | | | |
| **Purpose** | | | | | | | |
| The purpose of this study is to develop and analyze the non-invasive and non-contact vital signs monitoring method using radar and compare its accuracy with the invasive or contact vital signs monitoring method, which can be referred to as a standard inspection method for monitoring existing vital signs. | | | | | | | |
| **Background and Rationale** | | | | | | | |
| 1. Necessity   Monitoring of vital signs, such as respiratory rate and heart rate, has been developed so far as it is necessary for medical needs mainly for patients in a hospital environment, and various measurement methods have been developed. Recently, vital signs monitoring has increased in terms of disease and health care outside the hospital. In hospitals, invasive or contact methods are often used to ensure accuracy in environments such as neonatal or adult intensive care units. Vital signs are monitored in more ways. However, current methods of monitoring contact signs or invasive vitality in a hospital setting are at risk of several additional problems, such as opportunistic infection problems in patients with reduced immunity in the intensive care unit and skin detachment due to contact terminals in premature babies.  Recently, as a mother's age increases, high-risk pregnant women tend to increase. At the same time, despite the decline in fertility, the relative birth rate of premature babies is increasing. In addition to this premature infant, within 28 days of birth, newborns are admitted to the neonatal intensive care unit for a variety of causes, including capillary bronchitis, enteritis and sepsis. The need for 24 hour vital signs monitoring is high. So far, contact and invasive vital signs have been used to monitor these vital signs, but this method often shows false alarms and immature skin due to children's movements, and peeling and necrosis of skin at the site of attachment due to impaired vascular circulation . There is always the possibility that children, such as opportunistic infections, are at risk.  For this reason, conventional standardized monitoring in hospital patients, especially newborns involved in this study, demonstrates the limitations posed by intrusion and contact monitoring methods and new ways to monitor vital signs to overcome these limitations. The need to seek is increasing.   1. IR-UWB radar technology background  - New bio-signal and motion monitoring technology using IR-UWB radar technology   IR-UWB radar technology is a technology that uses an impulsive signal that occupies a wideband frequency and has a very short duration in the time axis, and has excellent resolution in the time axis, which is very useful for detecting minute movements due to breathing and heart rate seen during sleep.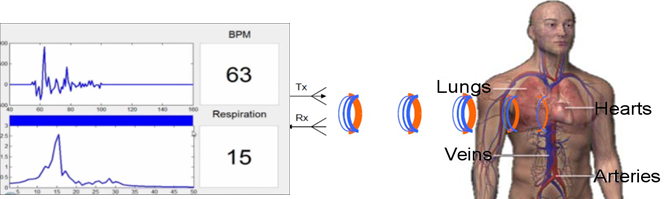  - Research related to the measurement of respiration rate and heart rate using the actual IR-UWB radar technology is being actively conducted in various parts of the world, and related product research is also being actively conducted.  - The IR-UWB radar-based monitoring technology can detect breathing, heart rate and movement, and apnea symptoms with a single sensor, and has the technical potential to measure blood pressure.  - The IR-UWB radar-based monitoring technology can detect breathing, heart rate and movement, and apnea symptoms with a single sensor, and has the technical potential to measure blood pressure.  - Unlike the existing attachment and wear sleep testing equipment, the sensor has a non-contact/non-invasive long-distance characteristic, so it is possible to increase user convenience and resolve rejection related to sleep monitoring.  - IR-UWB radar-based monitoring sensor can be manufactured in low cost and small size, so users can purchase and use it at a reasonable price.  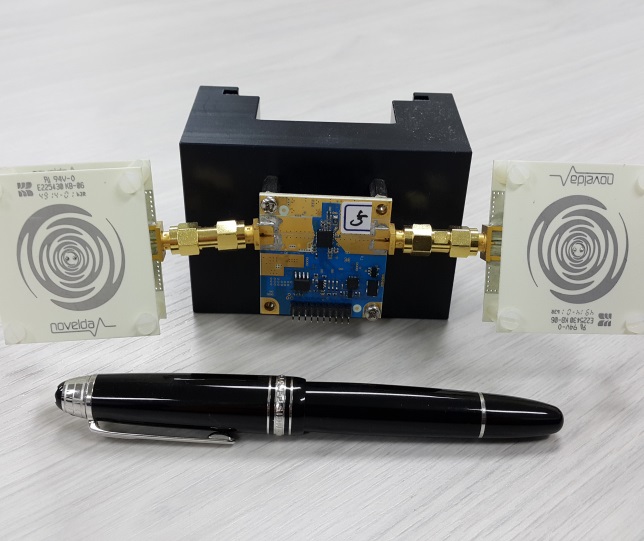  - IR-UWB radar has the technical possibility of extracting vital sign information for two or more people simultaneously with a single sensor.  - Non-contact vital sign monitoring is available in the hospital, and it can be used in a home rather than in a hospital, so it can be used by individuals with various conditions such as newborns, the elderly, patients, and people with disabilities.  - In relation to IR-UWB radar-based sleep monitoring with various features, Hanyang University of Technology team has secured the world's best technology, and research results of the research team's preliminary research are also posted on YouTube, a global video sharing site. The video was indirectly recognized for its performance to the extent that it was also introduced by chip maker NOVELDA.  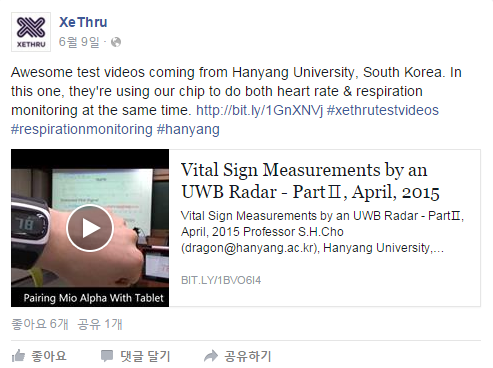  Therefore, compared to the existing traditional vital sign monitoring method through the invasive or contact method that is being performed in a real hospital environment at the present time, themethod using the IR-UWB radar is of clinical significance to develop technologies and devices that can obtain accurate biosignals in a variety of environments in a non-contact manner by checking their accuracy in the future. | | | | | | | |
| **Method** | | | | | | | |
| 1. Research content 2. Summary   The study is targeted at inpatients who are monitoring of vital signs (respiratory rate, heart rate, blood pressure) in Neontal Intensive Care Unit by the research team of professor Sung Ho Cho of College of Engineering, Hanyang University and the medical staff of Hanyang University Medical Center. The accuracy of the vital signs measured using the traditional method and the method using the IR-UWB radar were compared. The target group was selected for the term infants with a gestational period of 37 weeks or more admitted to the NICU for 18 months after passing the IRB.  The measurement index is the heart rate and respiration rate measured simultaneously with the IR-UWB radar and the traditional methods (ECG for heart rate / visual measurement, pulse oximeter, sensing device for respiratory rate).   1. Concrete method  - Clinical experimental environment - Cooperation with Hanyang University Medical Center & College of Medicine - To monitor vital signs in the NICU, an environment capable of measuring IR-UWB radar signals at the top of the incubator and basket (approximately 1.8 m) is built.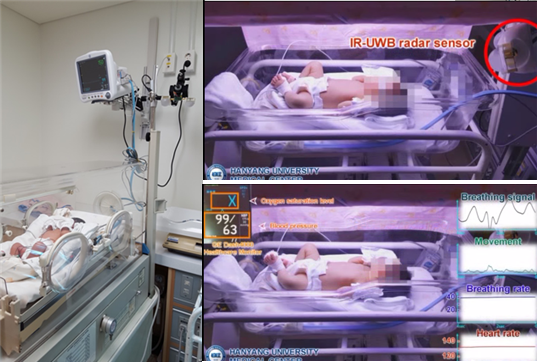   < Test bed environment established in the NICU >   - Improved respiratory rate / heart rate extraction algorithm for neonates   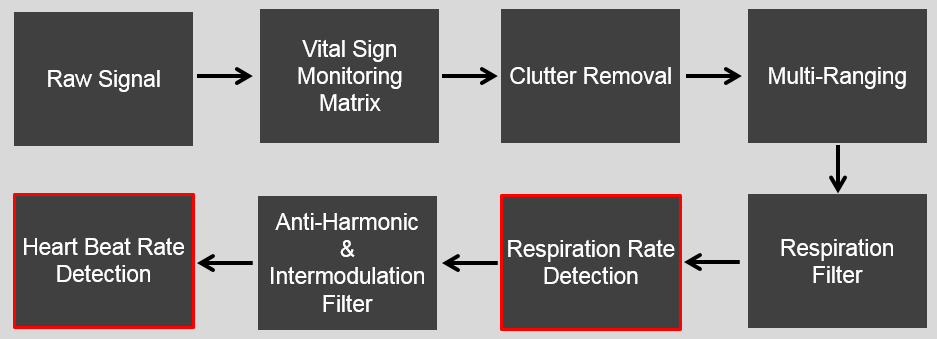  < Block diagram of algorithms for vital sign monitoring >   - In order to improve the stability of the respiratory rate measurement algorithm, respiratory rate information of the point with the highest reliability is extracted by using multiple points as candidate points in the IR-UWB radar signal.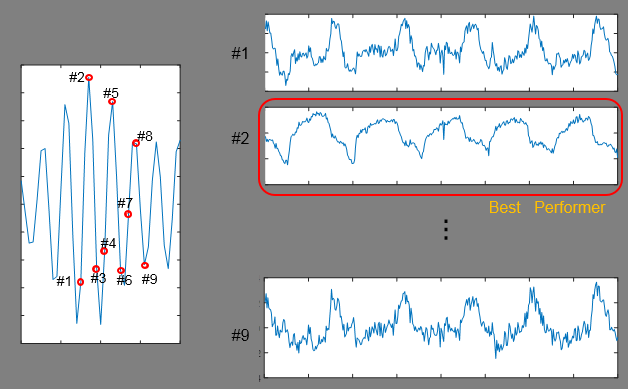   < Multi-point post-processing algorithm to improve respiratory rate measurement accuracy >   - Heart rate and respiratory rate measurement by IR-UWB radar   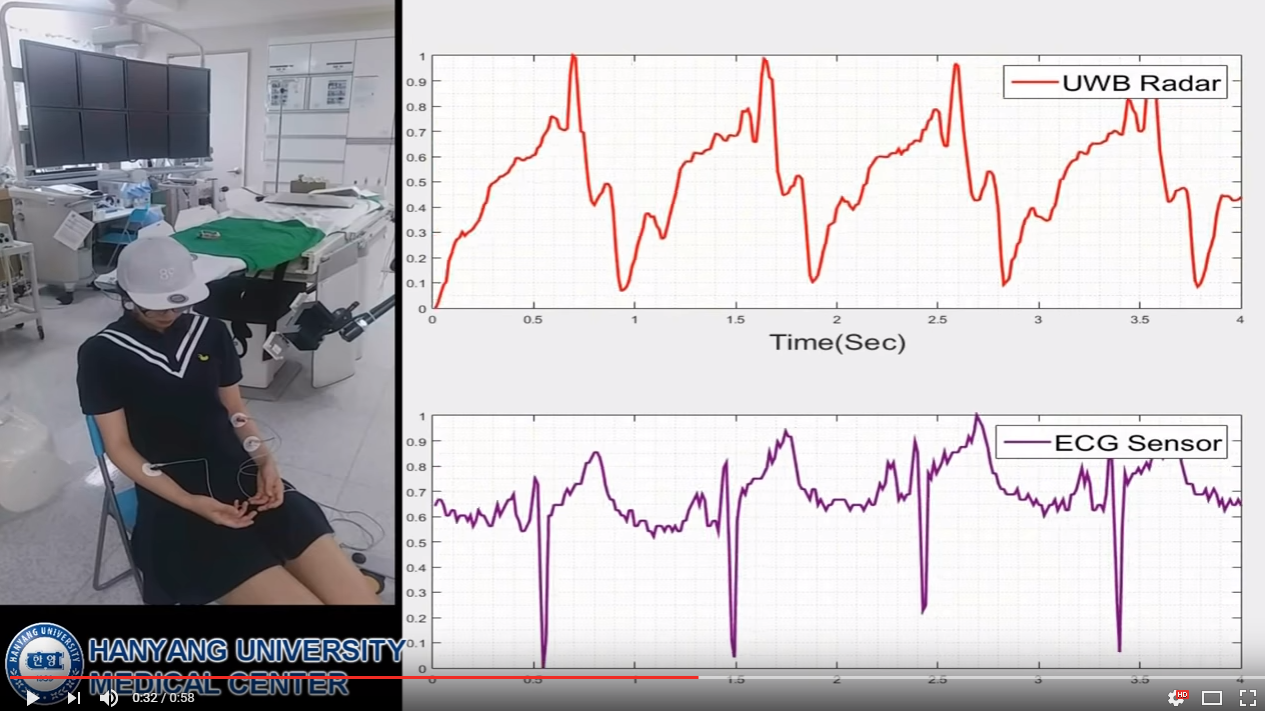  < Electrocardiogram and IR-UWB radar signal of a 26-year-old woman >   - Detailed plan of the research - Patients who have consented to the study are continuously measured for 3 days after being placed in a test bed built in the neonatal intensive care unit, and information about this is stored in a database. - In addition, record the results measured on the monitor in both cases at a fixed time (three times a day) on a case sheet. At this time, the presence and degree of movement of the infants are also recorded. - While conducting this study, patient monitoring is performed in an confined and isolated space in the NICU, and does not affect other patients in the NICU.  1. Expected effects  - Continuous monitoring for remote patients (non-invasive, non-contact) - Real-time alert enables quick emergency service in critical situations such as apnea - It is possible to monitor the vital signs of the elderly living alone or without a caregiver and without medical facilities to increase the work efficiency of public officials and to recognize and respond to high-risk patients early.  1. Research promotion plan 2. Strategy and Method  - Formation of research infrastructure through the establishment of a network of Hanyang University Medical Center and the radar research team of Hanyang University College of Engineering - Cooperation with professional medical staff - Verification through clinical trials  \| 1. 연도별 연 Research Schedule \| \| \| \| \| \| \| \| --- \| --- \| --- \| --- \| --- \| --- \| --- \| \| Year \| Contents \| Schedule \| \| \| \| Remarks \| \| 2017 / 2^nd^ half \| 2018 / 1^st^ half \| 2018 / 2^nd^ half \| 2019 / 1^st^ half \| \| 2017-8 \| Improvement of performance and function for vital signs monitoring \| ○ \| ○ \| ○ \|  \|  \| \| 2017-8 \| Hardware build \|  \| ○ \| ○ \|  \|  \| \| 2017-8 \| Software implementation \|  \| ○ \| ○ \| ○ \|  \| \| 2017-8 \| Reliability and Accuracy test \|  \| ○ \| ○ \| ○ \|  \|   **4. Research period**  Up to 2 years after passing the IRB (Institutional Review Board)  **5. Criteria for Enrolled Subjects ; Inclusion / Exclusion / Target number**  1) Inclusion criteria   1. Full term neonates requiring vital signs monitoring in NICU who have obtained consent   2) Exclusion criteria   1. Patients who do not agree to participate in the research 2. Sudden exacerbation of clinical symptoms and changes in vital signs that require discontinuation of the research   3) Protect vulnerable subjects  The subject of this study includes minors under the age of 19, and requires the consent of parents or other legal guardians. Therefore, in this study, for the protection of vulnerable research subjects, under 6 years of age, only the legal guardian of the research subject should be included in the research subject.  4) Target number of subjects  - calculated with Statistical analysis software PASS 2008  - The primary analysis is the inter-class correlation coefficient R (ICCR) between heart rate/respiratory rate measured via conventional contact and heart rate/respiratory rate measured by a non-contact method using an IR-UWB radar.  - As it is a test for vital signs, it should have high accuracy and assume Power = 0.90 and alpha = 0.01 for each indicator.   1. Heart rate (beats/min): 50 patients   The statistical method used in this study, the Interclass Correlation Coefficient method, is generally regarded as excellent inter-observer variability when the correlation coefficient (R) is 0.75 or more, but the subject of this research is about vital signs. Considering the point, it is judged that it should show higher accuracy.  Although the method using the IR-UWB radar is innovative in that it is a non-contact method, it should at least show a similar level of accuracy as the recently developed wearable heart rate monitoring device.  - Polar V800 heart rate monitor ; ICCR=0.976 (Gile et al, *Eur J Appl Physiol (2016) 116:563–57*)  - Apple watch ; ICCR=0.98 / Mio ALPHA ; ICCR=0.91 / Samsung Gear S=0.80 (Wallen et al, *PLoS ONE 2016, 11(5); e0154420*)  Given the above, ICCR in our study aims at 0.90 to 0.98. Therefore, if ICCR is <0.90, it will show low accuracy compared to the existing measurement method. When ICCR >0.98, it is assumed that it will show superior results than the existing measurement method. It is considered to be necessary, and considering that it is a pediatric subject that has a high possibility of dropping out of the subject and limiting the extraction of results, 50 targets, twice the number, are set as the target number of subjects.   1. Respiratory rate : 50 patients   According to a report by Bergese at al *(Anesth Analg 2017;124:1153–1159)*, the respiratory rate measured by Medtronic’s pulse oximetry (NellcorTM OxiMax N-600x Pulse Oximeter) showed an ICC of 0.94 in healthy people. In this study, the respiratory rate measured by the conventional respiratory rate measurement method and the ICC between the respiratory rate measured by the IR-UWB radar were determined to be at least similar to those measured by contact using a conventional pulse oximeter.  Accordingly, as the heart rate, ICCR is set to 0.90 to 0.98 and calculated to set 50 subjects as target subjects.  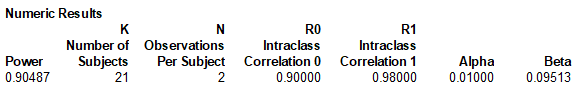  **6. Evaluation and Interpretation method (Statistical analysis)**  The evaluation criterion is to confirm the agreement between the heart rate/breathing measured by the IR-UWB radar and the conventional method, and the statistical analysis method mainly uses three methods.   1. Simple correlation, linear regression & residual plot 2. Interclass correlation coefficient R (ICCR) 3. Lin’s Concordance Correlation Coefficient R (CCC) | | | | | | | |
| **Research period** | | | | | | | |
| Up to 2 years after passing the IRB (Institutional Review Board) | | | | | | | |

- Research Ethical Considerations
  - Ethical management standards

In conducting this study, all participants participating in this study will perform ethically and scientifically in accordance with the procedures set forth in the Standards Work Guidelines and in compliance with the Helsinki Declaration, Bioethics and Safety Law, etc.

- - Instructions and Consent for the subject

Consent to the study manual and subject is documented separately, and if consent is given to the study, consent is provided to the subject along with a copy of the study agent and a description of the study agent.

- - Confidentiality

In this study, records that can identify the participants of the study are kept confidential, and personal information and personal information are not collected separately. Therefore, it is impossible to identify participants in the research process or in the subsequent publishing process. Data and files collected during the participants' consent and research process are thoroughly protected under the Berlin Data Protection Act and are used for research purposes only. Statistical analysis, publication or publication of research results does not include information that identifies the subject. All of this confidentiality and safety management is performed by research director, professor Hyun-Kyung Park.
