# Supplementary material for: Feasibility of non-contact cardiorespiratory monitoring using impulse-radio ultra-wideband radar in the neonatal intensive care unit
Source: PLoS One. 2020 Dec 28;15(12):e0243939. doi: 10.1371/journal.pone.0243939 (PMC7769476; doi:10.1371/journal.pone.0243939)
Supplement: Attachment — Submitted filename: Response to Reviewers.docx [file pone.0243939.s010.docx]

**Reviewer #1: This study investigates the feasibility of non-contact cardiorespiratory monitoring using impulse-radio ultra-wideband radar in the Neonatal Intensive Care Unit by comparing the measures with conventional electrocardiography (ECG)/impedance pneumography (IPG). I have several comments and questions for Statistical analysis.**

**1. Line 211, the abbreviation of SD must be defined the first time it is used.**

: Thanks for your comment. We have defined the term of SD in Line 204 at first instead.

**2. Please make it clear what is “the bias between the radar and the conventional measurements”. Does such “bias” equal to the difference between the radar and the conventional measurements?**

: Thanks for your detailed comment. We want to talk about the difference between the radar and the conventional measurement, so we have changed “bias” to “differences” as your comment in Line 211.

**3. I do not see any reports in this manuscript about intra-individual variances and inter-individual variances. How would you incorporate repeated measures in one-way ANOVA?**

: Thank you for your deep interest in statistically analysis. We used CCC to check the agreement of the measurements of the IR-UWB radar sensor and conventional measurements. Various statistical methods were considered to analyze the agreement between both methods, but since one-way ANOVA was not used, the content has been removed from the manuscript.

**4. According to data shown in S1 table, in Figure 5, the titles of the two boxes should be exchanged.**

: Thank you for your comments. As your comment, it is correct that the two boxes in Figure 5 have been exchanged. Including the comment below, we would revise Figure 5 accordingly.

**5. In figure 5, what do those vertical bars represent? One sample t-tests is not necessary because small difference from zero will get significant result when the sample size is large. To reflect the mean bias, 95% CI may be used.**

: Thank you for your comments. Figure 5 shows the t-test results for the difference between heart rate and respiratory rate of the IR-UWB radar sensor and the conventional measurements. Vertical bar means CI, and the figure is incorrect, so we corrected the figure rightly.

During the overall review of the manuscript submitted, there was a mistake in the radar device name used for data collection. We changed the product name to XK200 (Xandar Kardian, Delaware, USA) in Line 126 and Figure 1. Also, the website address related to FCC certification has expired, so we have attached a new address and revised it in Line 133. We’re sorry for such a mistake.
