# Supplementary material for: Feasibility of non-contact cardiorespiratory monitoring using impulse-radio ultra-wideband radar in the neonatal intensive care unit
Source: PLoS One. 2020 Dec 28;15(12):e0243939. doi: 10.1371/journal.pone.0243939 (PMC7769476; doi:10.1371/journal.pone.0243939)
Supplement: Attachment — Submitted filename: (2nd) Response to Reviewers.docx [file pone.0243939.s011.docx]

Review Comments to the Author

**Reviewer #2: Review of: Feasibility of non-contact cardiorespiratory monitoring using impulse-radio ultrawideband radar in the Neonatal Intensive Care Unit
Lee WH et al. PONE-D-20-21820R1**

**Lee et al report the ‘next step’ in development of a non-contact cardiorespiratory monitoring technology for heart rate and respiratory rate monitoring of newborn infants. This follows their pre-clinical report published last year (Park JY et al,** [**https://www.ncbi.nlm.nih.gov/pmc/articles/PMC6695386/**](https://www.ncbi.nlm.nih.gov/pmc/articles/PMC6695386/) **). It is well written, and provides information on a promising technology. They do a good job in describing the limitations and next steps before this technology moves closer to clinical application. As a non-statistician, my most significant question for this or a follow-on question is how reliable, valid and accurate the new technology is relative to the gold standard when analyzing high and low Heart Rates and respiratory rates? Can alarms be triggered? Would they be triggered too often, not often enough, or at an expected rate? Other minor comments/questions follow.**

**Introduction.
The authors might want to identify citations to justify the statement: “Repetitive replacement of electrodes and the twining wires around the arm or leg cause skin damage, infections due to skin layer breakdown, permanent scars, and circulatory disturbances, particularly in premature infants with fragile skin. There may even be a risk of hypothermia during procedures, which could cause circulatory disturbances, particularly in premature infants.

In addition to reference 2, the authors may want to cite a more recent reference on the impact of all the current leads in use on family interactions with their neonates (Bonner O, et al. 'There were more wires than him': the potential for wireless patient monitoring in neonatal intensive care. BMJ Innov. 2017;3(1):12-18. doi:10.1136/bmjinnov-2016-000145).**

: Thanks for your comments. As you advised, we have added the above reference to our manuscript (Reference 9).

**METHODS
Very minor, but in the location for MATLAB, the authors list the company location as “MathWorks, New York, MA, USA”). I think the headquarters of the company is Natick, MA, USA.**
: Thank you for your comments. We have modified the location of the company’s headquarter in Line 131.

**Very minor, line 176 of the revised manuscript w/ tracked changes, one word is misspelled: “...want to increase the Frames per sencod (FPS) of the radar to increase the quality of...” should be “...want to increase the Frames per second (FPS) of the radar to increase the quality of...”.**
: Thank you for your comments. The corresponding misspelled words was modified and corrected in line 178.
**I am not that statistically savvy, but would be interested in how well the correlations hold up at the lower and upper extremes of HR and RR. Are there specific statistical tests for measurement comparisons w/ gold standard technologies that accentuate the evaluation of the extremes? Maybe something like Figure 4 with HR and RR low, high and middle rates in place of the BW groupings would give a visual representation that I’m sure a wise statistician could translate more quantitatively.**

: Thank you for the comment. We have compared the bias levels among the 3 body weight groups through a graphical presentation and one sample t-test in the last manuscript. However, the more proper method to compare the bias levels among 3 groups would be one-way ANOVA (analysis of variance) with/without post-hoc tests such as Bonferroni or Tukey method. Therefore, we included the results of ANOVA tests among the 3 body weight groups in the revised manuscript (Figure 5A).

In response to your comment, we think that the bias levels between the radar and the conventional methods could also be compared among categories divided using HR and RR. Lin’s concordance correlation coefficients should not be used for the comparison because there were no established methods for the correction of the range restriction problem that must follow the categorization of the variable through dividing the data range. However, comparisons of the biases between two measurements could bypass this problem. We defined HR and RR > upper 5% and < lower 5% as the extreme values and divided the measurements into 3 categories as follow; Category 1: <5%, Category 2: 5~95% and Category 3: >95%. HRs were divided at 115 and 149 bpm and RRs were divided at 25 and 67 breaths/minute. We compared the bias levels between the two measurement methods among these 3 groups for both HR and RR using graphical presentations and one-any ANOVA (Figure 5B).

The biases between the two measurement methods were smaller in the category 2 (5%~95%) in both HR and RR and the radar measured HR and RR more frequently in the low HR and RR range and less frequently in the high HR and RR range.

In accordance with your comment, we revised the manuscript including these analysis results as Figure 5B.

**DISCUSSION
It’s one really good thing to pick up the normal range for HR and RR. How does the radar technology perform in picking up apnea and bradycardia? Could the authors in this (or a subsequent) paper tell the reader specifically about correlations between two techniques in alarms and abnormal readings on the low and high ends of both HR and RR?**

: Thank you for your comments. We are developing algorithms to detect apnea, arrhythmia and bradycardia using radar technology. However, in the NICU environment, the treatment comes first when the symptoms appear, so the low number of times apnea, arrhythmia, and bradycardia have occurred is early to verify the accuracy of detecting them. We will conduct an experiment to accurately detect and verify the accuracy of this part in further research.
